# Evaluating the Impact of a Risk Assessment System With Tailored Interventions in Germany: Protocol for a Prospective Study With Matched Controls

**DOI:** 10.2196/17584

**Published:** 2020-10-01

**Authors:** Marten Pijl, Jorn op den Buijs, Andreas Landgraf

**Affiliations:** 1 Collaborative Care Solutions Department Philips Research Eindhoven Netherlands; 2 Philips GmbH Hamburg Germany

**Keywords:** interventional study, personal emergency response system (PERS), population management, predictive modeling

## Abstract

**Background:**

With a worldwide increase in the elderly population, and an associated increase in health care utilization and costs, preventing avoidable emergency department visits and hospitalizations is becoming a global priority. A personal emergency response system (PERS), consisting of an alarm button and a means to establish a live connection to a response center, can help the elderly live at home longer independently. Individual risk assessment through predictive modeling can help indicate what PERS subscribers are at elevated risk of hospital transport so that early intervention becomes possible.

**Objective:**

The aim is to evaluate whether the combination of risk scores determined through predictive modeling and targeted interventions offered by a case manager can result in a reduction of hospital admissions and health care costs for a population of German PERS subscribers. The primary outcome of the study is the difference between the number of hospitalizations in the intervention and matched control groups.

**Methods:**

As part of the Sicher Zuhause program, an intervention group of 500 PERS subscribers will be tracked for 8 months. During this period, risk scores will be determined daily by a predictive model of hospital transport, and at-risk participants may receive phone calls from a case manager who assesses the health status of the participant and recommends interventions. The health care utilization of the intervention group will be compared to a group of matched controls, retrospectively drawn from a population of PERS subscribers who receive no interventions.

**Results:**

Differences in health care utilization and costs between the intervention group and the matched controls will be determined based on reimbursement records. In addition, qualitative data will be collected on the participants’ satisfaction with the Sicher Zuhause program and utilization of the interventions offered as part of the program.

**Conclusions:**

The study evaluation will offer insight into whether a combination of predictive analytics and case manager-driven interventions can help in avoiding hospital admissions and health care costs for PERS subscribers in Germany living at home independently. In the future, this may lead to improved quality of life and reduced medical costs for the population of the study.

**Trial Registration:**

Deutsches Register Klinischer Studien (DRKS), DRKS00017328; https://www.drks.de/drks_web/navigate.do?navigationId=trial.HTML&TRIAL_ID=DRKS00017328

**International Registered Report Identifier (IRRID):**

DERR1-10.2196/17584

## Introduction

With the worldwide increase in the elderly population [1], chronic diseases and associated health care utilization, such as costly emergency department visits and subsequent hospitalizations, are also on the rise. Elderly patients >75 years of age account for up to a third of ambulance transports in Germany [2]. Following worldwide trends, German elderly aged 65-74 years, on average, show a 50% increase in health care expenditures compared to the 50-64 age bracket. For the elderly aged 75 and over, the increase is 100% [3]. Preventing avoidable emergency department visits and admissions in elderly patients is becoming a global priority [4,5] since emergency hospitalizations are particularly distressing for the elderly and have been associated with adverse events such as hospital-acquired infections, loss of functional independence, and falls [6].

A Personal Emergency Response System (PERS) service consists of a help call button worn by the subscriber on the wrist or as a pendant. When a PERS subscriber requires assistance, a press of the button will establish a live connection to a personal response agent in a response center. The agent is then able to triage the event, and when applicable, assist the subscriber, either over the live voice connection, or by contacting a relative, informal caregiver, care provider, or emergency services.

The main benefit of a PERS service is the reassurance that help will always be available in case of an emergency, such as a fall or respiratory issues. Previous studies in the United States (US) [7,8] have shown that PERS data can be used to develop prediction models of decline in patient status. Such models thus provide early warning signs of impending emergencies and can be used by case managers to provide timely intervention [9]. Currently, it is unknown whether the combination of predictive modeling and case manager interventions is also effective in Germany, with a different health care system and potentially a different PERS subscriber population compared to the US. Whereas the US health care system is characterized by vertically integrated care providers and a strong private pay mentality, the German system is fragmented with very limited ability of health insurances to create and enforce care pathways.

Risk prediction can play an important role in preventing future hospitalization by allowing an opportunity for timely interventions and can play an instrumental role in reducing health care costs and utilization [10-13]. In the Sicher Zuhause (Safe at Home) study, the risk scores are used by a case manager (along with other data such as demographics, medical alert history, or medical conditions) to determine participants potentially at risk of hospitalization in a German PERS subscriber population. The case manager may then proactively contact the at-risk participant, and if needed, intervene at an early stage, before hospitalization becomes inevitable.

### Objectives

The aim of the Sicher Zuhause study—a collaborative effort by Philips, the Techniker Krankenkasse (TK), ServiceCall AG, and the German Red Cross (DRK)—is to evaluate whether the combination of risk scores determined through predictive modeling and targeted interventions offered by a case manager can result in a reduction of hospital admissions and health care costs in a German PERS subscriber population. The primary study aim is to determine whether there is a difference in the number of hospitalizations between the intervention group and the control group over the 8-month intervention period.

In addition, the study aims to assess several secondary outcomes; these are the impact on the number of hospitalizations leading to admissions, the number of days admitted to a hospital, the number of ambulatory visits, the hospital-related costs, and the overall health care costs. Further, qualitative data collected through questionnaires will be used to assess the participants’ satisfaction with the PERS devices used in the study and with the Sicher Zuhause program as a whole.

## Methods

### Study Design and Overview

The Sicher Zuhause study is a prospective interventional study with an intervention arm of 500 participants, which is compared to a matched population derived from historical PERS subscribers. The study tracks the health care utilization and expenditure of the participants over 8 months. During this period, the participants in the intervention arm may receive calls from one of the case managers in the study with the offer to discuss the participant’s health status and care needs. Based on this discussion, the case manager may recommend one or more interventions.

**Figure 1:**
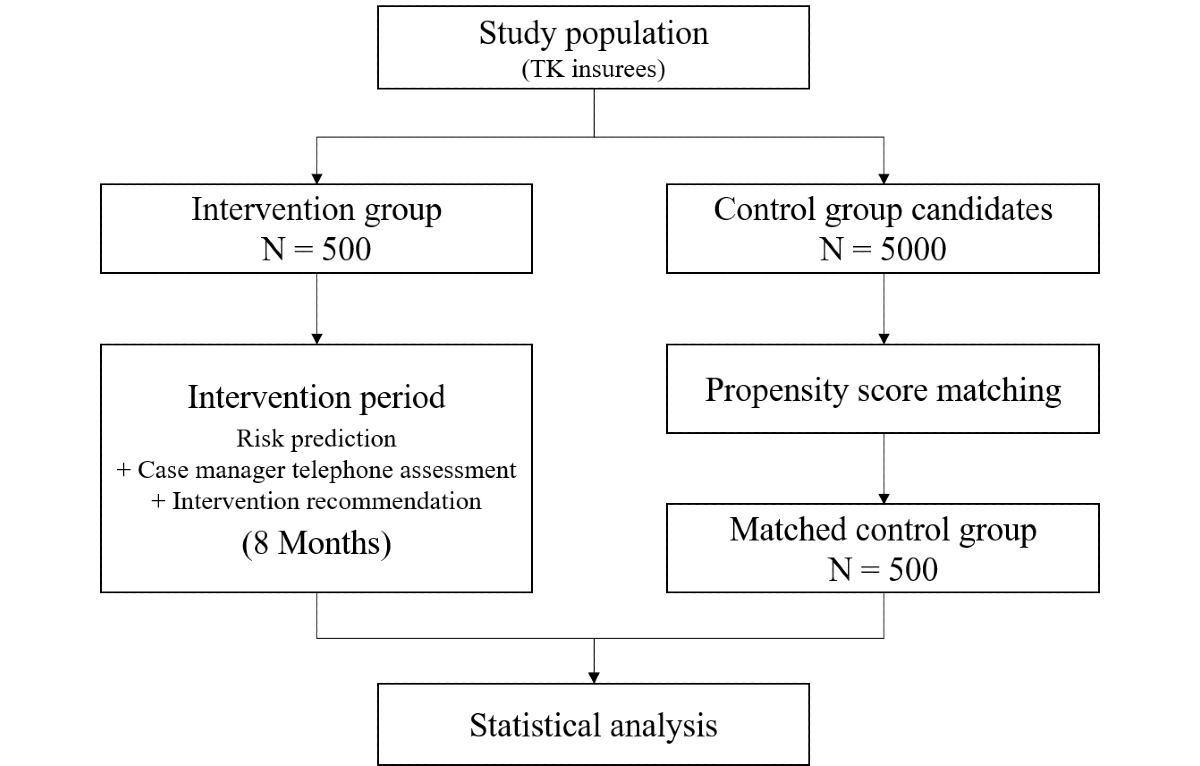
Study design.

The matched population serving as a control group does not actively take part in the study and is instead derived from the historical records of PERS subscribers. This population will be matched with the intervention arm to be as similar as possible compared to the intervention group using propensity score matching. An overview of the study design is shown in Figure 1.

### Participants and Selection Criteria

Study participants will be recruited from a pool of individuals who are insured by the TK—a statutory health insurance organization based in Hamburg. To be included in the study, participants must be eligible for PERS reimbursement, have a “care level” (Pflegegrad) as defined under the German health care system of at least 1 and at most 4, and be of at least 18 years of age.

Exclusion criteria for the study are severe cognitive impairments, severe hearing impairments, severe motor impairments, mental disorders, or other medical conditions that prohibit participation due to the inability to provide consent or to communicate with the case manager over the phone. Participants who are expected to be outside the home for a significant amount of time (due to planned hospital admission, for example) of the intervention period will be excluded. This applies to any single period of over 3 weeks or a cumulative period of over 2 months.

### Enrollment

Potential study participants will be recruited through one of several ways:

1. New PERS customers or customers of the nursing service of ServiceCall or the DRK who are insured at the TK will be asked if they are interested in learning about the study.
2. Potential participants may be made aware of the study through articles published by the TK (for example, in their member magazine).
3. Potential participants may be identified from among existing TK customers. These potential participants may be contacted directly by the TK by phone or mail.

Identified individuals will be sent an information letter and informed consent. Individuals will be enrolled in the study after returning the signed informed consent letter.

### Study Sites

The participants will use the PERS service in their own homes. During the study, the participants will make use of one of two response centers operated by ServiceCall (Kassel, Germany) and DRK (Düsseldorf, Germany).

### Risk Score Calculation

During the study, the participants’ risk will be assessed by a predictive model of hospital transport in the next 30 days. This predictive risk model was developed on a retrospective data set of more than 8000 deceased German PERS subscribers using a methodology similar as described in [7], which is used by the Philips CareSage predictive analytics engine in the United States. The predictive model achieved an area under the receiver operator characteristic curve of 0.75.

The predictive model calculated the participants’ risk based on a combination of medical alert pattern data collected by the call centers, as well as demographic information and self-reported medical conditions. The provided risk score is relative to the average risk of the population used to develop the model (eg, a risk of 2 indicated an estimated risk two times higher than the average). The distribution of the risk scores will be monitored during the study in case there are major differences between the study population and the population used to develop the model.

### Intervention

**Figure 2:**
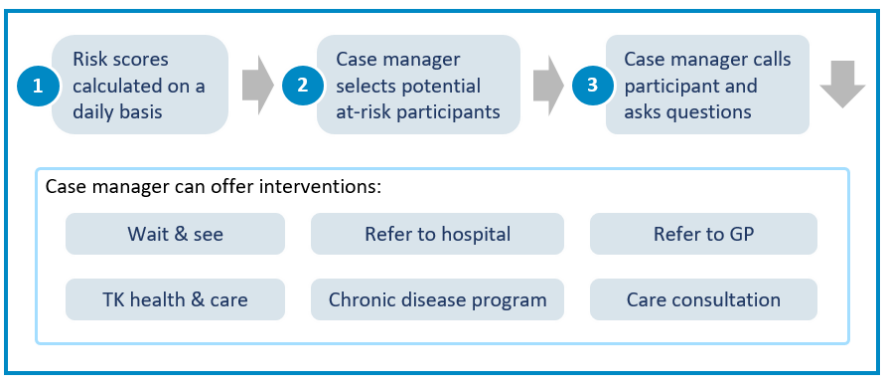
Intervention protocol.

Throughout the study, risk scores will be calculated daily and provided to a case manager. While risks are predicted for 30 days, daily calculations of the risk score allow for new information to be taken into account as soon as possible. Based on the height and trend of the risk scores, the history of response center interactions and demographic data, the case manager decides which participants to contact to further inquire about their health status and care needs (Figure 2).

When a case manager contacts a participant, they will first evaluate the participant’s health status through a triage protocol. Based on this conversation, the case manager may conclude to offer one or more interventions. As part of this study, the case manager may recommend:

1. No further action is needed at this time.
2. To plan a general practitioner visit.
3. To contact emergency medical services.
4. To enroll in one of the TK’s existing health and care offers. These can, for example, include training programs on nutrition, a fall prevention course, or cardiovascular training.
5. To schedule a care consultation.

Interventions by the TK are offered free of charge. If one or more interventions are recommended, the case manager will schedule a follow-up call with the participant after 14 days. During the follow-up call, the case manager will inquire if the participant followed the suggested actions, and if so, what was the outcome (eg, referral to a specialist or dietary recommendations).

At the start of the study, participants will also receive a welcome call from the case manager to obtain a baseline measure of the participant’s health status and already plan interventions if necessary. The participant may ask any questions that remain regarding the study.

### Data Collection

The main source of data for the analysis of health-related outcomes will be the reimbursement records collected by the TK. These contain the type of care and the associated costs of care received by the participants during the intervention period. The expected time for these records to become available to the TK is approximately 1 month for hospital and PERS-related costs, and up to 9 months for ambulatory and specialist costs. At the end of the study period, participants will be asked to complete a questionnaire on health care utilization during the intervention period.

Response center data, including records of interactions with the personal response agents, demographics, and reported medical conditions, will also be collected during the intervention period for risk score calculation and the evaluation of the study. The case managers will also document the recommended interventions and actions taken by the participant during the follow-up call after an initial recommendation.

#### Data Analysis

The analysis of the primary outcome, the number of hospitalizations, will be based on a comparison of reimbursement records between the intervention arm and a matched population acting as a control group. Propensity score matching will be used to obtain a comparable control group [14,15] using a large pool of population characteristics (over 100 variables), which include the age at enrollment, gender, geographic location, care level, and presence or absence of common medical conditions. The intervention arm of 500 participants will be compared to an equal-sized matched control group of 500, determined by propensity score matching out of an initial pool of approximately 5000 candidates. Differences between groups will be analyzed through statistical testing, using the binomial rate test for the number of admissions and the t-test for differences in costs.

Analysis of the qualitative data provided by questionnaires will focus on evaluating the participants’ satisfaction with the Sicher Zuhause program overall, and with the individual components such as the case manager interactions and the offered interventions. This will be done through descriptive statistics such as the mean and standard deviations of the satisfaction and helpfulness measured on Likert scales.

The qualitative data will be used to provide insights into to outcomes of the primary objectives and may assist in the improvement of the CareSage predictive model.

#### Sample Size Calculation

A study with a comparable population by Coleman et al [16] reported a reduction for hospital visits over a 180-day period to 23% in the invention group versus 32% in matched controls. Based on these proportions, 80% power, and a type I error (alpha) of 0.05, at least 386 participants in each group are needed. Accounting for an estimated lost to follow-up of 15%, this increases to at least 454 participants. The sample size for the intervention arm was therefore set at 500.

#### Ethical Considerations

The Sicher Zuhause study will be conducted in accordance with the most recent version of the Declaration of Helsinki. The study has been assessed and approved by the internal board of ethics of Philips Research (Internal Committee of Biomedical Experiments), and has been reviewed and approved by the Western Institutional Review Board (WIRB, tracking number 20182221).

All data collected in the study will be maintained in compliance with the General Data Protection Regulation privacy directive and the German data protection law. Data will be pseudonymized where possible and kept encrypted both in transit and at rest.

## Results

The enrollment of participants began mid-2019 and is expected to be complete by end of 2020. Enrollment is expected to take longer than usual based on the number of new PERS subscribers over time in the geographical area covered by the response centers in the study. Data collection is expected to be completed by summer of 2020. Initial results are expected to be available in fall of 2021.

## Discussion

This protocol describes a study that aims to determine if the combination of risk scores determined through predictive modeling and interventions offered by a case manager can result in a reduction of hospital admissions and health care costs in a German PERS subscriber population. The rapidly aging population with multiple comorbidities presents numerous challenges to effective care management, and therefore, alternatives to hospital and institutional care are needed to optimize health care costs and improve patient outcomes [17]. In the future, the results of this study can lead to better health outcomes for the population described.

This study has a few limitations. First, it does not include a randomized design, and the use of a retrospective control group may lead to selection bias. In the study, this concern is partially addressed by the use of propensity score matching. Second, the effect of the risk prediction and the interventions cannot be easily separated. As such, the study aims to evaluate the combination of the two, where the predictive model helps to identify the right subset of participants to whom interventions should be offered. Further, analysis of the qualitative data can help to provide insights into the performance of the predictive model and the effectiveness of the interventions. Another option would be to include additional study arms; however, due to the expected recruitment rate, this is likely not feasible.

A third limitation is that it might be difficult to capture data on health care utilization that may be initiated outside of the PERS service. Capturing emergency department visits and hospitalizations is important because the study population is a high health care utilization group that is at risk for emergency transport, and patients are typically taken to the nearest medical center for emergency care services. However, self-reported data from patients may help mitigate some of these challenges.

## Abbreviations

DRK: German Red Cross (Deutsches Rotes Kreuz)
DRKS: German clinical trials register (Deutsches Register Klinischer Studien)
PERS: personal emergency response system
TK: Techniker Krankenkasse
US: United States
